# Sexual behaviour of men that consulted in medical outpatient clinics in Western Switzerland from 2005-2006: risk levels unknown to doctors?

**DOI:** 10.1186/1471-2458-10-528

**Published:** 2010-09-02

**Authors:** Françoise Dubois-Arber, Giovanna Meystre-Agustoni, Jeannin André, Kim De Heller, Pécoud Alain, Patrick Bodenmann

**Affiliations:** 1 Institute of Social and Preventive Medicine (IUMSP), University Hospital Centre and University of Lausanne, Lausanne, Switzerland; 2 Department of Ambulatory Care and Community Medicine, University Hospital of Lausanne, Lausanne, Switzerland; 3 Vidy-Source Outpatient Clinic, Lausanne, Switzerland

## Abstract

**Background:**

To determine male outpatient attenders' sexual behaviours, expectations and experience of talking about their sexuality and sexual health needs with a doctor.

**Methods:**

A survey was conducted among all male patients aged 18-70, recruited from the two main medical outpatient clinics in Lausanne, Switzerland, in 2005-2006. The anonymous self-administered questionnaire included questions on sexual behaviour, HIV/STI information needs, expectations and experiences regarding discussion of sexual matters with a doctor.

**Results:**

The response rate was 53.0% (N = 1452). The mean age was 37.7 years. Overall, 13.4% of patients were defined as at STI risk - i.e. having not consistently used condoms with casual partners in the last 6 months, or with a paid partner during the last intercourse - regarding their sexual behaviour in the last year. 90.9% would have liked their physician to ask them questions concerning their sexual life; only 61.4% had ever had such a discussion. The multivariate analysis showed that patients at risk tended to have the following characteristics: recruited from the HIV testing clinic, lived alone, declared no religion, had a low level of education, felt uninformed about HIV/AIDS, were younger, had had concurrent sexual partners in the last 12 months. However they were not more likely to have discussed sexual matters with their doctor than patients not at risk.

**Conclusion:**

Recording the sexual history and advice on the prevention of the risks of STI should become routine practice for primary health care doctors.

## Background

Health authorities and medical associations recommend that general practitioners (GPs) should be active in STI prevention [1-3], and articles that offer guidance on the issue have been published in medical journals [4,5]. In Switzerland, individual advice on STI prevention by GPs is an essential component of the National AIDS Prevention Strategy and it is expected that they identify patients at risk for STIs and offer them prevention advice[6]. However, the current literature suggests that GPs' discussions with patients on sexuality issues and related prevention remain insufficient [7-11]. A lack of discussion on sexual matters with GPs was reported since the 1990 s, both in the US [12-14] and in Europe [10] in surveys among patients of primary care physicians [15]. Observations of GPs' prevention skills with standardized patients confirmed that questions regarding sexuality are asked least often [16]. Studies conducted among physicians indicated similar observations: studies in the 1990 s suggested that talking about AIDS or sexuality [17], or routinely providing an assessment of sexual behaviour was rare among physicians [7,8,10].

Currently, we know very little about the sexual behaviour and associated STI risks of patients that consult with GPs. Yet this information is important for assessing patient STI prevention needs and assessing the effects of implementing systematic STI prevention activities by GPs. In Switzerland, as in many countries, the sexual behaviour of the general population (aged 17-45 in Switzerland) is periodically assessed through phone surveys in the realm of behavioural surveillance [18-20]. For example, in 2000, 21% of men 17-30 years old and 10% of men 31-45 years old had had occasional partner(s) in the past 6 months, and 35% and 45%, respectively, had not consistently used condoms with their partners [21]. However, we do not know whether these findings can be directly applied to patients consulting in general medical practice clinics.

The aim of our study was to determine male outpatients attenders' sexual behaviours - particularly those considered to increase the risk of acquiring an STI - as well as expectations and lifetime experience of talking about their sexuality and prevention needs with a doctor. Data were collected from the two main outpatient city clinics in 2005-2006 in Lausanne, Switzerland, one of them being an important university centre for GPs training.

## Methods

A consecutive recruitment survey was conducted among all male patients aged 18-70 who attended the two main outpatient city clinics in Lausanne, Switzerland, from October 2005 to February 2006. The two main outpatient clinics were: 1) the university department of ambulatory care and community medicine, a primary setting for GPs training in a multicultural context, that included 5 clinics (general internal medicine consultation, medical emergencies, travel medicine/vaccines, HIV anonymous testing, and dental), and 2) a private medical outpatient clinic with an emergency department and five GP practices. Patients were excluded if they were severely ill, psychologically disturbed, illiterate, or could not understand one of the questionnaire languages (Albanian, English, French, German, Italian, Portuguese, Serbo-Croatian, Spanish, and Turkish). The anonymous self-administered questionnaire was provided by clerical staff before the medical visit (in travel/vaccines clinics and emergency clinics) or by the doctor (in general consultation and dental clinics) or nurse (in the HIV anonymous testing clinic) after the consultation, with a brief explanation of the study. Patients mailed the completed questionnaires directly to the research team. The total number of consultations with male patients, their age and nationality, and the reasons for not offering the questionnaire were recorded. The questionnaire contained questions on the following topics (the specific questions, the coding frame, as well as the recall periods for assessment are shown in Table 1 and the questionnaire can be seen in Additional file 1):

**Table 1:** Comparison of patients at risk versus patients not at risk, in % (Lausanne, 2005-2006)

Variables	All	Risk	No risk	p value^§^
*Recruitment site (N = 1431)*				
General/internal medicine and emergencies*	40.0	37.5	40.4	< 0.001

Dental clinic	3.2	2.1	3.4	
	
Travel clinic	31.6	19.3	33.6	
	
Anonymous HIV testing	18.0	36.5	14.3	
	
Private practitioner**	7.8	4.7	8.3	

**Socio-demographic characteristics**				

*Median age (N = 1413)*	34	31	35	
	
*Mean age*	37.6	33.5	38.4	< 0.001

*Education (N = 1418)*				

Compulsory school	7.3	6.3	7.5	0.100
	
Vocational training	26.7	34.0	25.6	
	
Technical school	21.5	18.3	22.0	
	
University	44.4	41.4	44.9	

*Nationality (N = 1413)*				

Swiss	69.6	64.4	70.5	0.080
	
European	19.7	22.5	19.2	
	
African	5.5	8.9	5.0	
	
Other	5.2	4.2	5.3	

*Religion (N = 1410)*				

Protestant	33.1	24.2	34.5	0.011
	
Catholic	36.9	36.8	36.9	
	
Muslim	5.1	4.7	5.2	
	
Other	3.5	5.3	3.3	
	
Without	21.3	28.9	20.2	

*Employment status (N = 1413)*				

Working	77.9	82.2	77.3	0.098
	
Not working	17.0	15.7	17.2	
	
Pensioned	5.1	2.1	5.6	

*Living situation (N = 1411)*				

Living with partner	53.3	20.6	58.3	< 0.001
	
Living alone with a partner elsewhere	24.1	32.8	22.7	
	
Living alone	22.6	46.6	18.9	

**Sexual behaviour**				

Age at first sexual intercourse*** (N = 1387): under 16 years old	18.3	25.0	17.3	0.011

Ever had same sex sexual intercourse (N = 1416) yes	11.4	21.9	9.8	< 0.001

Had sexual concurrency in the last 12 months (N = 1276) yes	26.6	69.8	20.4	< 0.001

Median N of partners in the last 12 months (N = 1390) Mean N of partners in the last 12 months	1 2.9	4 6.4	1 2.4	< 0.001

Any STI symptoms in the last 12 months**** (N = 1370) yes	8.8	16.0	7.7	< 0.001

**HIV test, information and discussion**				

Ever been tested for HIV(N = 1402) yes	65.8	77.5	63.9	< 0.001

Feel well/rather well informed about HIV/AIDS (N = 1352) yes	92.3	86.4	93.2	0.001

Feel well/rather well informed about other STI (N = 1392) yes	43.2	33.3	44.7	0.003

Wish to be asked questions about sexual history by the doctor ***** (N = 1412) Yes/rather yes	90.9	92.1	90.8	0.546

Ever discussed sexual issues with a doctor (N = 1413) yes	61.4	64.9	60.8	0.277

Ever been advised on how to avoid STI by a doctor (N = 1412) yes	37.4	46.6	36.0	0.005

* General consultation and emergencies in the public clinic + emergencies in the outpatient private clinic

** The five GPs in the private outpatient clinic

*** No definition of the term "sexual intercourse" was given to the patient

**** "During the past 12 months, have you experienced any pain when urinating, any discharge from your penis, or ulcers on your genitals"

***** "Would you like your doctor to ask you this type of question (on your sexual history) in order to give you advice that is better suited to your circumstances"

§ Pearson's Chi-square, except for mean age and mean number of partners in the last 12 months, where the t-test was used

- socio-demographic characteristics

- sexuality: sexual orientation, age at first intercourse, numbers and types of partners (stable, occasional, paid), condom use with each type of partner and during the last intercourse, sexual concurrency (having had sexual intercourse with different partners over the same period during the last 12 months), and experience of STI symptoms

- level of information about STIs reported by the patient

- expectations and experiences regarding sexual history taking, sexual advice, and HIV testing.

Most of the questions on sexual behaviour were taken from general population surveys conducted as part of the Swiss behavioural surveillance system [18], in order to allow comparison with the last survey conducted in 2000. The questionnaire was pre-tested with experts in each of the languages for cultural adaptations. A variable was constructed to analyze the potential risk of exposure to an STI, defined as non use of condoms with a non primary partner. This choice reflected the way of conceptualizing "risk behaviour" in the Swiss HIV/AIDS policy: sexual risk is essentially defined by non use of condoms during intercourse, any other situation or characteristic - such as having sex with a paid partner or having several partners - preceding the choice of using or not condoms is not defined as risk per se but only as potential risk [18,22]. Consequently "patients at risk" were defined as those that had not consistently used condoms with casual partners in the last 6 months, or those that had not used a condom during last intercourse with a paid partner in the last 12 months (the two variables determining condom use with occasional, and paid partner(s) available in the surveillance questionnaire). Patients at risk were compared with those not at risk in a bivariate analysis using Pearson's chi-2 and t-test with a 95% confidence interval.

We used multiple independent variables logistic regression to identify the characteristics associated with patients at risk. The variables included: age in years (over/under 41 years old), recruitment site (recruited from the HIV anonymous testing clinic/all other departments), declared religion (none/any religion), nationality (Swiss citizenship/other), education (vocational training or less/further education), living situation (alone/with a partner), age at first intercourse (less than 16/16 and over), ever had same sex intercourse (yes/no), number of partners in the last 12 months (continuous), sexual concurrency in the last 12 months (yes/no), ever tested for HIV (yes/no), any STI symptoms in the last 12 months (yes/no), feel informed on AIDS (well/not well), feel informed on STIs (well/not well), ever discussed at least one sexual issue with a doctor (yes/no), ever been counselled by a doctor on how to avoid STIs (yes/no). A total of 298 respondents (21.5%) were missing information on one or more of these variables and were excluded from this analysis by listwise deletion. These excluded respondents did not differ from those included in the regression either on the main socio-demographic variables (age, marital status, education) except nationality (slightly less Swiss citizens in the excluded), or on the variable patient at risk.

This study has been approved by the Ethics Committee of the University Hospital Centre of the Canton of Vaud (CHUV), Lausanne, Switzerland.

## Results

The overall response rate was 53.0% (N = 1452), from 41.7% in the internal medicine/emergency clinics of both outpatients clinics (GIM/E) to 79.2% in the travel clinic. However, respondents did not differ from the eligible population as a whole to which the questionnaire was given, in age (mean 37.6 years vs 37.7). Furthermore, Swiss citizens were only slightly overrepresented compared to non-Swiss respondents (69.6/65.1). The non-response rate for each question was low, between 0.4 to 10.8% (question on concurrency).

Forty percent of the total sample was recruited from the general internal medicine/emergency clinics of both outpatient clinics (GIM/E), 31.7% from the travel clinic, 17.3% from the anonymous HIV testing clinic, 8.3% from the 5 GP clinics, and 3.4% from the dental clinic. Among these, 1431 patients had had sexual intercourse and were retained for the subsequent analysis.

We expected and found significant differences across different clinics with regard to: response rate (from 41.8% in emergencies to 79.2 in the travel clinic), mean age (from 30.7 in the HIV testing clinic to 45.7 in the five GPs clinics), nationality (from 59.4% of Swiss citizens in GIM/E clinics to 79.1 in the travel clinic), sexual behaviours (see below). Therefore the recruitment site was introduced as independent variable in the multivariate analysis.

Behaviours that posed a potential risk of exposure to STIs were reported by a high proportion of patients. Sexual intercourse with occasional partner(s) was reported by 32.4% of the patients (from 24.4% in the travel clinic to 58.2% in the HIV testing clinic, p < 0.001), and condom use with these partner(s) by 57.8% (from 50.7% in the HIV testing clinic to 67.9% in the five GP clinics, ns); With regard to age classes for comparison with the last general population survey (2000) on similar questions (see introduction), the proportion of people having had one or more occasional partners in the last 6 months in our study was 43.0% among the 17-30 years old (21% in the general population in 2007) and 33.4% among the 31-45 years old (10% in the general population in 2007); respectively 42.0% and 42.2% of them had not used condoms consistently (35% and 45% in the general population in 2000). The proportion of patients with occasional partners was substantially higher than among the male general population, and condom use with occasional partner(s) was lower among the younger patients in our sample.

Having paid for sex in the last twelve months was reported by 12.8% (from 8.0% in the GPs clinics to 15.2% in the HIV testing and the GPs clinics, p = 0.442), and condom use at last paid intercourse was reported by 94.2% (from 91.3% in the GIM/E clinic to 100% in the dental clinic, ns). Sexual concurrency was reported by 26.6% (from 19.6 in the travel clinic to 52.5% in the HIV testing clinic, p < 0.001).

Overall, 13.4% of the patients (from 8.0% in the GP's clinics to 15.2% in the HIV testing clinic, p < 0.001) were defined as patients at risk for STIs. This category included 323 patients reporting occasional partners and no paid partners, 118 patients reporting occasional partners and paid partners, and 53 patients reporting only paid partners.

90.9% of patients reported that they wished their doctor would ask them questions about their sexuality in order to receive counselling, but only 61.4% had previously had this experience and overall only 37.4% had ever received STI prevention counselling.

Patients at risk were younger than those patients who were not identified as at risk, and were more likely to have been recruited in the HIV testing clinic (Table 1). There was no difference between the two groups in terms of education and employment status. Patients at risk were more likely to be of a nationality other than Swiss, to live alone, and to declare no religion. In terms of sexuality, patients at risk tended to have had an earlier sexual debut, more sex partners, more same sex intercourse experience. In addition, in the last 12 months, a higher proportion of patients at risk had had sexual concurrency and had had STI symptoms than had those patients not at risk.

Patients at risk felt less well informed about HIV/AIDS and other STIs than other patients and were less likely to have been tested for HIV. Although a higher proportion of patients at risk had been advised by a doctor on how to avoid STIs, they were not more likely to ever have discussed sexual matters with a GP as compared to those patients not at risk.

In a multivariate analysis, we found that the strongest risk factors for patients at risk were being under the age of 41 (OR 3.98), living alone (OR 3.12), and to have concurrent sexual partners (OR 5.09) (Table 2).

**Table 2:** Variables associated with patients at risk (Lausanne, 2005-2006)

*Variables*	OR	Sig	IC 95%
*Recruitment site*			
other clinics	1		
HIV anonymous testing clinic	**1.69**	.026	1.06 - 2.71

*Age*			
Continuous (in years)	**1.04**	.008	1.01 - 1.08
dichotomized: under 41	**3.98**	.003	1.59 - 9.94

*Education*			
Technical school or university	1		
Compulsory school or vocational training	**1.73**	.013	1.12 - 2.66

*Nationality*			
Swiss	1		
Other	1.12	.605	.72 - 1.74

*Religion*			
Any religious affiliation	1		
None declared	**1.82**	.010	1.15 - 2.89

*Living situation*			
Living with a spouse/partner	1		
Living alone with or without a spouse/partner elsewhere	**3.12**	.000	1.92 - 5.08

*Age at first intercourse:*			
16 years old and over	1		
Under 16 years old	1.23	.413	.74 - 2.03

*Ever had same sex sexual intercourse:*			
No	1		
Yes	.87	.638	.49 - 1.53

*Any STI symptoms in the last 12 months*			
No	1		
Yes	**1.85**	.049	1.00 - 3.42

*N of partners in the last 12 months (continuous)*	1.03	.087	0.99 - 1.07

*Concurrency in the last 12 months*			
No	1		
Yes	**5.09**	.000	3.20 - 8.10

*Ever been tested for HIV*			
Yes	1		
No	1.32	.293	.78 -2.24

*Feel informed about HIV/AIDS*			
Well/rather well	1		
Not very well/not well	**.40**	.010	.20 - .80

*Feel informed about other STI*			
Well/rather well informed	1		
Not very well/not well	.85	.511	.53 - 1.36

*Would like to be asked questions about sexual history by doctor*			
Yes/very much	1		
Not really/no	1.18	.668	.54 - 2.61

*Ever discussed sexual issues with a doctor*			
No	1		
Yes	1.28	.325	.78 - 2.11

*Ever been advised on how to avoid STI by a doctor*			
No	1		
Yes	1.05	.832	.64 - 1.71

## Discussion

In this study, we determined sexual behaviours of male patients attending two city outpatients clinics. In particular, we compared specific items among the same age classes between our sample and a representative sample of the general population from a survey conducted in Switzerland in 2000. A sizeable proportion of patients that consulted in outpatient clinics reported sexual behaviours that may increase the risk of exposure to an STI, this proportion was higher than the one observed among men in the general population. Patients at risk, i.e. those having not used condoms with a non primary partner, possessed some socio-demographic characteristics that differentiated them from patients not at risk: they were younger, lived alone, had a lower level of education, had no religious affiliation, and felt less informed about HIV/AIDS. Only one behavioural factor - sexual concurrency - was identified for patients at risk which was independently associated with risk in the multivariate analysis; this factor suggests that there may be the potential for "sexual bridging" between populations [23-25]. On the other hand, patients at risk did not differ from patients not at risk in their willingness to be asked about their sexual history by a doctor nor did they differ from patients not at risk in lifetime experience of discussing sexual issues with a doctor.

In our study, approximated one respondent in seven was found to be a patient at risk for STIs, by not having used a condom with a non primary partner. It is noteworthy that this estimate of patients at risk is conservative, given that our definition of "at risk" was very restrictive, related only to the inconsistent use of condoms. Other potential risk situations, including sexual concurrency (which we found among more than one patient in four) or having occasional partners (which we found among approximately one patient in three), are more frequently reported. As expected, the level of risk was higher among patients from the HIV testing clinic, although it was not negligible among patients from the other clinics. Very few studies have analyzed sexual behaviours in patients seen in GP practices; those who did also pointed to frequent high risk sexual behaviour [26]. In an Australian study, about a quarter of the patients aged 18-50 had been in "nonmutually monogamous heterosexual relationships" with low rates of condom use [27]. We found that the proportion of people that had occasional partners was much higher among our respondents than among the general population. Although the rate of non condom use with occasional partners was more similar between the two populations, it was higher among the outpatient city clinic younger patients in our sample than among the general population in the 2000 study. Previous research suggest that individuals who are less socially integrated (e.g. migrants) and who have no family doctor tend to be overrepresented in outpatient city clinics [28]; such individuals may have different lifestyles or live in more unstable relationships [29] that might explain the differences from the general population with regard to sexual behaviour.

We found that less than two thirds of the patients had ever had the occasion to discuss sexual matters with a doctor. Patients at risk felt less informed about HIV/AIDS than patients not at risk; however, they were not more likely to have ever discussed sexual matters with a doctor than patients not at risk; in other terms, risk patients were not given more attention as we would expect. This is disturbing, especially when we consider that the vast majority of our respondents - at risk or not - reported that they would like to be asked about sexuality by their doctors in order to receive advice on STI prevention. The willingness of patients to be asked questions about sexuality has been reported in other studies [30,31]. The discrepancy between patients' wishes and actual lifetime experience of discussing sexual matters with a doctor suggest that barriers are still present on the side of doctors. Therefore it is important that doctors, in particular future GPs on university training, be aware of the level of patients' risk behaviours and of patients' expectations, in order to improve their sexual history taking and prevention advice delivery. Recent studies among doctors report higher rates of involvement in recording sexual history and routinely discussing STI prevention [32]. In 2004, 81% of US clinicians in seven specialties that commonly provide STI diagnoses, treatments, and prevention services reported recording sexual histories [33]. In Switzerland, 63% of primary care physicians in 2002 reported that they would systematically assess HIV risk behaviour with their young adult patients [11]. An increasing number of STI diagnosed in general practice has been observed in the UK and may also reflect an increasing involvement of GPs with sexual health [34].

This study had limitations, including response biases often present in sexually sensitive research. The response rate differed across the different clinics. Patients with a very active sex life may have been more prone to answer a questionnaire on this topic; although previous research suggests that this may not be the case [35]. Although the response rate was not high, it was satisfactory in a study where it was not possible to send reminder. Moreover, it is likely that the patients with a low literacy level did not mention that they were illiterate and were given the questionnaire; these patients were counted as non-participants when in fact they were not eligible; their number is not known. However, we believe that these limitations do not seriously affect the main findings, in particular the comparison between the patients at risk and those not at risk. Another limitation is the discrepancy between the relatively short time periods (six and twelve months) for reporting behaviours and the long time period (ever) to report experience of a discussion with a doctor on sexual matters. It may very well be that some patients at risk may not have had the opportunity to have a sexual health-related discussion with a doctor during the period when they were exposed to risk. However, about 75% of adults in Switzerland consult a doctor yearly [36] and we also considered that, to a certain extent, current risk sexual behaviour is a marker of past behaviour. Finally, our results should be interpreted with caution when applying them to other city outpatients or GP clinics, because they may differ with different mixes of services.

## Conclusions

Our study suggests that sexual behaviours that put patients at risk of contracting STIs are common among patients of outpatient city clinics in Switzerland; we also found that patients expect and want to be asked questions about their sexual behaviour; recording the sexual history and offering advice on STI prevention should become a routine practice for primary health care doctors.

## Competing interests

The authors declare that they have no competing interests.

## Authors' contributions

The study was directed by FDA. Study and instruments were designed by FDA, GMA, AJ. GMA directed the data collection, KDH, AP, PB participated in the data collection. GMA and AJ conducted the analyses. FDA wrote the article, AJ and GMA participated in the writing. All authors read and approved the final manuscript.

## Pre-publication history

The pre-publication history for this paper can be accessed here:

http://www.biomedcentral.com/1471-2458/10/528/prepub

## Supplementary Material

Additional file 1 **Questionnaire: Sexually transmitted diseases and the need for prevention advice among patients**. Click here for file
