# Supplementary material for: Sexual behaviour of men that consulted in medical outpatient clinics in Western Switzerland from 2005-2006: risk levels unknown to doctors?
Source: BMC Public Health. 2010 Sep 2;10:528. doi: 10.1186/1471-2458-10-528 (PMC2939648; doi:10.1186/1471-2458-10-528)
Supplement: Additional file 1 — Questionnaire: Sexually transmitted diseases and the need for prevention advice among patients. [file 1471-2458-10-528-S1.PDF]

Institute of Social and Preventive Medicine
Bugnon 17, 1005 Lausanne

Director: Professor Fred Paccaud

Sexually transmitted diseases and the need for prevention advice among patients

Please note: the questionnaire is anonymous

How to fill out the questionnaire?
Please tick the circle beside the answer that best corresponds to your situation.
Unless indicated otherwise, please tick only one circle.

Thank you very much for taking the time to participate!

<u>Note for men who have relations with other men:</u>
For the sake of simplicity, we have chosen to refer consistently to female partners (e.g. wife, girlfriend). For the purposes of the questionnaire, these terms can also refer to male partner or boyfriend. We apologise for this, and thank you in advance for your understanding.

For many people, AIDS is a major cause for concern. How much do you know about this disease?

1 Please indicate whether the AIDS virus can be transmitted in the following ways:

(Please give an answer per line)	Yes	No	Don't know
In public toilets	O1	O2	O3
By injecting drugs with a syringe previously used by someone else	O1	O2	O3
Drinking from the same glass as a person with the AIDS virus	O1	O2	O3
Unprotected sexual intercourse	O1	O2	O3
When a woman with the AIDS virus gives birth	O1	O2	O3

Doctors need to know their patients sufficiently well to be able to treat them correctly. They ask their patients a variety of questions, for example on their medical history or on whether they smoke. They may also enquire about the patient's sexual history.

2 Would you find it normal for a doctor to ask you questions on your sexual history?

O1 Yes O2 Rather yes O3 Rather no O4 Not at all

3 Would you like your doctor to ask you this type of question in order to give you advice that is better suited to your circumstances?

O1 Yes O2 Rather yes O3 Rather no O4 Not at all

4 Generally speaking, when would be the best moment to talk about these issues?

O1 From the first appointment, as part of the series of lifestyle-related questions that the doctor generally asks

O2 At a later stage, when the doctor and patient know each other better

O3 Never

5 Would you be embarrassed if your doctor asked you this type of question?

O1 Yes O2 Rather yes O3 Rather no O4 Not at all

This section of the questionnaire concerns your own experience of the questions asked (or not) and advice given (or not) by your doctor concerning protection against the AIDS virus and other sexually transmitted diseases.

6 Which of the following issues has a doctor already spoken to you about?

(Please give an answer per line)

	Yes	No
Your sexual history in general	O1	O2
The number of sexual partners you have had	O1	O2
Protection against sexually transmitted diseases	O1	O2
Protection against unwanted pregnancy (contraception)	O1	O2
The gender of your partners	O1	O2
Previous history of sexually transmitted diseases	O1	O2

7 Has a doctor already advised you on how to avoid sexually transmitted diseases, including the AIDS virus?

O1 Yes O2 No

8 If yes, what did you think of his advice?

O1 Helpful O2 Rather helpful O3 Rather unhelpful O4 Unhelpful

9 How well are you informed about AIDS?

O1 Well informed O2 Rather well informed O3 Rather poorly informed O4 Poorly informed

10 How well are you informed about other sexually transmitted diseases (e.g. syphilis)?

O1 Well informed O2 Rather well informed O3 Rather poorly informed O4 Poorly informed

11 Do you need information on the following subjects?

(Please give an answer per line)

	Yes	No
The transmission of AIDS and other sexually transmitted diseases	O1	O2
Protection against AIDS and other sexually transmitted diseases	O1	O2
The treatment of AIDS and other sexually transmitted diseases	O1	O2

The following section of the questionnaire deals with your sexual history. Should you find any questions too personal, please feel free not to give an answer.

12 How old were you when you had sexual intercourse for the first time?

☐☐ Years old

○ I have yet to have sexual intercourse ➔ *(If no, please go to the information box that Question* 35)

13 Since you have been sexually active, have you ever used a condom during intercourse?

○1 Yes ○2 No

14 Did you use a condom the last time you had sexual intercourse?

○1 Yes ○2 No

15 Have you ever had sexual intercourse with a man?

○1 Yes ○2 No

16 How many sexual partners have you had over the last 12 months?

Number of partners ☐☐

17 At the present time or over the last 12 months, have or had you a stable sexual partner (wife, girlfriend)?

○1 Yes ○2 No

18 If yes, did you use condoms with your stable sexual partner?

○1 Always ○2 Sometimes ○3 Never

The following section of the questionnaire deals with 'casual sexual intercourse', that is 'unplanned' sex, short-term sexual relations or one-night stands, for which no payment was made.

Please note: the questions in this section relate only to events over the past six months.

19 **During the past 6 months, have you had one or more casual sexual partners?**

O1 Yes O2 No

20 **Did you use a condom with these partners?**

O1 Always O2 Sometimes O3 Never

21 **During the last 12 months, did you have sexual intercourse with different partners over the same time period?**

O1 Yes O2 No

The following section of the questionnaire deals with sexual relationships with people that are paid or given gifts in return for sexual intercourse.

22 **Have you ever accepted payment or gifts in return for sexual intercourse?**

O1 Yes O2 No

23 **Since you have been sexually active, have you ever paid or offered a gift in return for sexual intercourse?**

O1 Yes O2 No ➔ *(If no, please go to the information box that precedes Question 34)*

24 **How many times during the past 12 months?**

Number of times

25 **With how many individuals during the past 12 months?**

Number of individuals

This section of the questionnaire deals with your last sexual experience with a person whom you paid or to whom you offered a gift in return for intercourse.

26 **When did this last happen?**

O1 Within the last 12 months
O2 More than one year ago

27 **How did you meet the person whom you paid or to whom you offered a gift in return for sexual intercourse?**

O1 In the street
O2 Through classified ads or Internet ads for a massage parlour
O3 In a nightclub, cocktail bar or disco etc.
O4 In a club (e.g. for swingers)
O5 Through classified ads or Internet ads for escorts

28 **In which country did this take place?**

O1 In Switzerland
O2 Outside Switzerland

29 **Who was the last person you paid or to whom you offered a gift in return for sexual intercourse?**

O1 A woman
O2 A transvestite or transsexual
O3 A man

30 **Was the last person whom you paid or to whom you offered a gift in return for sexual intercourse a drug user?**

O1 Yes
O2 No
O3 Don't know

31 **Did you use a condom?**

O1 Yes
O2 No

32 **At the time of your last sexual experience with a person whom you paid or to whom you offered a gift in return for intercourse, were you also having sexual intercourse with another individual(s)?**

O1 Yes
O2 No

33 **If yes, did you use condoms with these other individuals?**

O1 Always
O2 Sometimes
O3 Never

A number of diseases can be transmitted during sexual intercourse. This section of the questionnaire deals with your sexual health.

34 During the past 12 months, have you experienced any pain when urinating, any discharge from your penis or ulcers on your genitals?

O1 Yes   O2 No

35 Have you ever taken an AIDS test?

O1 Yes   O2 No

36 How many AIDS tests have you taken to date?

Number of times

37 In what year did you have your last AIDS test? (please give an approximate answer, if you cannot remember the exact year)

Year

38 What were the results of your last test?

O1 Not infected with the AIDS virus
O2 Infected with the AIDS virus
O3 Don't know
O4 Do not wish to say

39 Have you ever injected drugs?

O1 Yes   O2 No

We have reached the end of the questionnaire. The following questions deal with essential information needed for our study. Please note, from the answers given, we are not able to identify the person who completed the questionnaire.

40 How old are you?

Years old

41 Which country or geographical region do you come from?

O1 Switzerland
O2 European Union
O3 Other Eastern European country, including Russia
O4 Asia
O5 North Africa or Middle East
O6 Sub-Saharan Africa
O7 North America
O8 Central or South America
O9 Other

42 How long have you been in Switzerland?

O1 Less than one year   O2 For one year or more

43 Which type of work and residence permit do you have?

O1 Swiss nationality
O2 C permit (settlement permit)
O3 B permit (residence)
O4 F permit (temporary admission)
O5 N permit (asylum seeker)
O6 Other or no permit
O7 Tourist visa

44 What are your current living circumstances?

O1 Living with your wife or girlfriend
O2 Living alone, but you have a wife or girlfriend who lives elsewhere
O3 Living alone and not in a stable relationship

45 What is your marital status?

O1 Single
O2 Married
O3 Separated or divorced
O4 Widowed
O5 Civil solidarity pact

46 What religion are you?

O1 Protestant
O2 Catholic
O3 Muslim
O4 Jewish
O5 Orthodox
O6 Other (please state) ______________________________
O7 None

47 What was the last education you received?

O1 Statutory schooling (completed or not)
O2 Apprenticeship
O3 Grammar school, baccalaureate, higher vocational college
O4 University, University of Applied Sciences

48 Are you in employment?

O1 Yes   O2 No

You have reached the end of the questionnaire.
Thank you very much for your valuable help!

We would also like to carry out short and anonymous interviews with people who have recently had several sexual partners. If you are interested, please call Giovanna Meystre-Agustoni on 021 314 72 91.
